# Defective CD4^+^CD25^+ ^regulatory T cell functioning in collagen-induced arthritis: an important factor in pathogenesis, counter-regulated by endogenous IFN-γ

**DOI:** 10.1186/ar1500

**Published:** 2005-01-28

**Authors:** Hilde Kelchtermans, Bert De Klerck, Tania Mitera, Maarten Van Balen, Dominique Bullens, Alfons Billiau, Georges Leclercq, Patrick Matthys

**Affiliations:** 1 Laboratory of Immunobiology, Rega Institute for Medical Research, Katholieke Universiteit Leuven (KULeuven), Leuven, Belgium; 2 Laboratory for Experimental Immunology, Department of Pathophysiology, Faculty of Medicine, Katholieke Universiteit Leuven (KULeuven), Leuven, Belgium; 3 Department of Clinical Chemistry, Microbiology and Immunology, Ghent University Hospital, Ghent, Belgium

**Keywords:** arthritis, autoimmunity, interferon-γ, regulatory T cells

## Abstract

Mice with a deficiency in IFN-γ or IFN-γ receptor (IFN-γR) are more susceptible to collagen-induced arthritis (CIA), an experimental autoimmune disease that relies on the use of complete Freund's adjuvant (CFA). Here we report that the heightened susceptibility of IFN-γR knock-out (KO) mice is associated with a functional impairment of CD4^+^CD25^+ ^T_reg _cells. Treatment of wild-type mice with depleting anti-CD25 antibody after CFA-assisted immunisation with collagen type II (CII) significantly accelerated the onset of arthritis and increased the severity of CIA. This is an indication of a role of T_reg _cells in the effector phase of CIA. IFN-γR deficiency did not affect the number of CD4^+^CD25^+ ^T cells in the central and peripheral lymphoid tissues. In addition, CD4^+^CD25^+ ^T cells isolated from naive IFN-γR KO mice had a normal potential to suppress T cell proliferation *in vitro*. However, after immunisation with CII in CFA, the suppressive activity of CD4^+^CD25^+ ^T cells became significantly more impaired in IFN-γR-deficient mice. Moreover, expression of the mRNA for Foxp3, a highly specific marker for T_reg _cells, was lower. We further demonstrated that the effect of endogenous IFN-γ, which accounts for more suppressive activity in wild-type mice, concerns both T_reg _cells and accessory cells. Our results demonstrate that the decrease in T_reg _cell activity in CIA is counter-regulated by endogenous IFN-γ.

## Introduction

The adaptive immune system uses various potent effector mechanisms for the elimination of foreign pathogens. Because these mechanisms are potentially damaging to the host, an essential feature of the immune system is its ability to distinguish self from non-self antigens and to develop tolerance to the former. With regard to T cell tolerance, the immune system has evolved several strategies. Most autoreactive T cells are eliminated during (primary) maturation in the thymus, a process described as negative selection, resulting in central T cell tolerance. Autoreactive T cells that escape negative selection will nevertheless be prevented from being activated as they are confronted with auto-antigen in the periphery. Several mechanisms have been proposed to account for this peripheral tolerance. One of those is suppression by a subset of T cells that express both CD4 and CD25. Evidence for the important role of these cells is overwhelming [1]. For example, when CD4^+ ^T cells isolated from peripheral lymphoid tissues of normal mice are depleted of CD4^+^CD25^+ ^T cells and injected into *nu*/*nu *mice, the recipients develop a high incidence of organ-specific autoimmune disease [2]. Co-transfer of the CD4^+^CD25^+ ^population prevents the induction of disease. CD4^+^CD25^- ^and CD4^+^CD25^+ ^T cells are therefore often designated as, respectively, T_eff _and T_reg _cells. CD4^+^CD25^+ ^T_reg _cells are generated in the thymus. Their development is directed by relatively high-avidity interactions between the TCR and self-peptide ligands [3-5]. The CD4^+^CD25^+ ^T_reg _cell population constitutes 5 to 10% of the mature CD4^+ ^cell population in the adult thymus and the peripheral lymphoid tissue and blood.

*In vitro*, CD4^+^CD25^+ ^T_reg _cells inhibit polyclonal T cell activation [6,7]. The suppression is mediated by a cytokine-independent, cell contact-dependent mechanism that requires activation of the CD4^+^CD25^+ ^cells via the TCR with specific antigen [8]. However, once stimulated, they are competent to suppress in an antigen-independent manner. Although the exact mechanism by which T_reg _cells exert their regulatory function is still unknown, there are indications that interaction of transforming growth factor-β (TGF-β) with its receptor [9-11], inhibition of IL-2 production [6] or downregulation of co-stimulatory molecules on antigen-presenting cells [12] could be involved.

T_reg _cells have proved to be important in various animal models of autoimmune diseases. Administration of anti-CD25 antibody *in vivo *induces organ-localised autoimmune diseases [13]. Inoculation of CD4^+ ^T cells depleted of CD25^+ ^cells in *nu/nu *mice results in autoimmune diseases such as gastritis, thyroiditis and insulitis [2]. Thus, transfer of T_reg _cells prevents autoimmune gastritis after neonatal thymectomy, and inhibits gastritis induced by H/K ATPase-reactive effector T cells [14]. MBP-specific CD25^+^CD4^+ ^T cells prevent spontaneous autoimmune encephalomyelitis in TCR-transgenic mice deficient in the recombination activating gene RAG-1 [15]. Similarly, CD4^+^CD25^+ ^T_reg _cells suppress central nervous system inflammation during active experimental autoimmune encephalomyelitis [16].

Collagen-induced arthritis (CIA) is a well-described animal model for rheumatoid arthritis. The disease is induced in genetically susceptible DBA/1 mice by immunisation with collagen type II (CII), and both T cell and B cell autoimmune responses are required for its development [17-19]. IFN-γ receptor knock-out (IFN-γR KO) mice have been found to suffer an accelerated and more severe form of CIA [20-23]. Moreover, knocking-out of the IFN-γ gene makes genetically resistant strains of mice susceptible to CIA [24,25]. These data indicate that deletion of the IFN-γ response somehow disrupts an endogenous protective mechanism against CIA.

Morgan and colleagues [26] have recently demonstrated that CD25^+ ^T_reg _cells are important in the pathogenesis of CIA. In the present study we confirmed the importance of T_reg _cells in the pathogenesis of CIA by rendering wild-type DBA/1 mice deficient in T_reg _cells by depleting anti-CD25 antibodies. Anti-CD25-treated mice developed a significantly more severe arthritis, comparable to the disease course in IFN-γR KO mice. Thus, we proposed that the higher susceptibility of IFN-γR KO DBA/1 mice to CIA might be ascribed to defects in the production (differentiation and homeostasis) or function of these CD4^+^CD25^+ ^T_reg _cells. We therefore determined the numbers of T_reg _cells in central and peripheral lymphoid organs of IFN-γR KO and wild-type mice. We further investigated whether T_reg _cells of IFN-γR KO mice have defects in the ability to suppress TCR-induced *in vitro *proliferation of CD4^+^CD25^- ^T_eff _cells.

## Materials and methods

### Mice and experimental conditions

The generation and the basic characteristics of the mutant mouse strain (129/Sv/Ev) with a disruption in the gene coding for the α-chain of the IFN-γ receptor (IFN-γR KO) have been described [27]. These IFN-γR KO mice were backcrossed with DBA/1 wild-type mice for 10 generations to obtain the DBA/1 IFN-γR KO mice used in the present study. The homozygous IFN-γR KO mice were identified by PCR as described [23]. Wild-type and IFN-γR KO DBA/1 mice were bred in the Experimental Animal Centre of the University of Leuven. The experiments were performed in mice 6 to 10 weeks old, but in each experiment the mutant and wild-type mice were age-matched within 5-day limits. The male : female ratio was kept between 0.8 and 1.3 in each experiment group, unless otherwise mentioned. All animal experiments were approved by the local ethical committee (University of Leuven).

### Induction and clinical assessment of arthritis

Native chicken CII (Sigma-Aldrich, St Louis, MO, USA) was dissolved at 2 mg/ml in PBS containing 0.1 M acetic acid by stirring overnight at 6°C and emulsified in an equal volume of complete Freund's adjuvant (CFA; Difco Laboratories, Detroit, MI, USA) with added heat-killed *Mycobacterium butyricum *(0.5 mg/ml). IFN-γR KO and wild-type mice were sensitised with a single intradermal injection at the base of the tail with 100 μl of the emulsion on day 0. From day 0 after immunisation, mice were examined for signs of arthritis five times a week. The disease severity was recorded with the following scoring system for each limb: score 0, normal; score 1, redness and/or swelling in one joint; score 2, redness and/or swelling in more than one joint; score 3, redness and/or swelling in the entire paw; score 4, deformity and/or ankylosis.

### Media, reagents and antibodies

All cells were grown in RPMI 1640 (Bio Whittaker Europe, Verviers, Belgium), supplemented with 10% heat-inactivated FCS (Gibco, Paisley, UK), penicillin (100 IU/ml; Continental Pharma, Brussel, Belgium), streptomycin (100 μg/ml; Continental Pharma), 2 mM L-glutamine, 10 mM Hepes (Gibco), 0.1 mM nonessential amino acids (ICN, Asse Relegem, Belgium), 1 mM sodium pyruvate (Gibco) and 50 μM 2-mercaptoethanol (Fluka, AG, Switzerland).

Anti-CD25 IL-2Rα monoclonal antibody was produced by hybridoma PC61 in an INTEGRA CELLine CL1000 (Elscolab, Kruibeke, Belgium) and is a rat IgG1 antibody. The hybridoma supernatant was purified by Protein G-Sepharose chromatography (Amersham Biosciences, Roosendaal, The Netherlands) for administration *in vivo*.

The hamster monoclonal antibody, directed against the mouse CD3 complex, was prepared from the culture supernatant of 145-2C11 hybridoma cells [28]. The antibodies were purified by affinity chromatography with Protein A-Sepharose (Amersham Biosciences). Batches of anti-CD3 antibody were tested for endotoxin content with the *Limulus *amebocyte lysate QCL-1000 kit (Bio Whittaker) and were found to contain less than 3 ng/ml endotoxin.

### Cell purification

Lymph nodes (axillary, inguinal and mesenteric) and spleens were harvested from mice 6 to 8 weeks old. Lymph nodes and spleens were gently cut into small pieces and passed through cell strainers (Becton Dickinson Labware, Franklin Lakes, NJ, USA). Red blood cells were lysed by two consecutive incubations (5 and 3 min at 37°C) of the suspension in NH_4_Cl (0.83% in 0.01 M Tris-HCl, pH 7.2). Remaining cells were washed, resuspended in cold PBS and counted. Lymph node preparations were then enriched for CD4^+ ^T cells with the Mouse T cell CD4 Subset Column Kit (R&D systems, Abingdon, UK). To purify CD4^+^CD25^+ ^and CD4^+^CD25^- ^cells, the enriched CD4^+ ^T cells were incubated for 20 min at 4°C with FITC-conjugated anti-CD25 and phycoerythrin (PE)-conjugated anti-CD4 antibodies (10 μg per 10^8 ^cells) in PBS containing 2% FCS. They were sorted by flow cytometry on a FACS Vantage (Becton Dickinson, San Jose, CA, USA). The resultant purity of the CD4^+^CD25^- ^population was 99%, whereas the purity of the CD4^+^CD25^+ ^population varied from 96% to 99%. Alternatively, CD4^+ ^T cells were labelled with PE-conjugated anti-CD25 monoclonal antibody, followed by incubation with magnetic-activated cell sorting (MACS) anti-PE beads (CD25 Microbead Kit; Miltenyi Biotec, Bergisch Gladbach, Germany). CD4^+^CD25^+ ^T cells were selected on an LS column in a magnetic field and the flow-through was collected as CD4^+^CD25^- ^T cells. After removal of the column from the magnetic field, CD4^+^CD25^+ ^T cells were flushed out by a plunger. The purity of the CD4^+^CD25^- ^population was 99% and the purity of the CD4^+^CD25^+ ^population varied from 90% to 95%.

T cell-depleted spleen suspensions were prepared by MACS (Miltenyi Biotec) and used as accessory cells (ACs). For MACS separation, the cell suspension was magnetically labelled with CD90 (Thy1.2) microbeads and passed through a CS separation column, placed in a magnetic field. The unlabelled CD90^- ^cells ran through.

### Flow cytometry

Single-cell suspensions (5 × 10^5 ^cells) were incubated for 15 min with the Fc-receptor-blocking antibodies anti-CD16/anti-CD32 (CD16/CD32; BD Biosciences Pharmingen, San Diego, CA, USA). Cells were washed with PBS containing 2% FCS and stained with the indicated FITC-conjugated antibodies (0.5 μg) for 30 min, washed twice and incubated for 30 min with the indicated PE- or biotin-conjugated antibodies. For the biotin-conjugated antibodies, a third staining step with streptavidin conjugated with peridinin chlorophyll *a *protein (PerCP) was performed. After washing, propidium iodide (Sigma-Aldrich) was added at a final concentration of 4 μg/ml to distinguish dead cells from living cells. Biotin-conjugated anti-CD25 (7D4), FITC-conjugated anti-CD25 (7D4), FITC-conjugated CD69 (H1.2F3), PE-conjugated anti-CD4 (RM4-5) and PerCP-conjugated streptavidin were purchased from BD Biosciences Pharmingen. FITC-conjugated anti-CD62L (MEL-14) and anti-CD44-FITC (IM7.8.1) were from CALTAG Laboratories (Burlingame, CA, USA).

For intracellular staining with anti-CTLA-4-PE (UC10-4F10-11; BD Biosciences Pharmingen), 10^6 ^cells were first labelled with anti-CD25-FITC as described above. Then, cells were fixed, permeabilised and stained with anti-CTLA-4-PE using the Cytofix/Cytoperm™ Kit (BD Biosciences Pharmingen) according to the recommendations of the manufacturers.

Flow-cytometric analysis was performed on a FACScan flow cytometer with Cell Quest software (Becton Dickinson).

### Proliferation assays

CD4^+^CD25^- ^cells (5 × 10^4 ^per well) were cultured in U-bottomed 96-well plates (200 μl) with ACs (5 × 10^4 ^per well, 30 Gy γ-irradiated or treated with mitomycin-C (Sigma-Aldrich)), 3 μg/ml anti-CD3 and the indicated numbers of CD4^+^CD25^+ ^cells for 48 hours at 37°C in 7% CO_2_. Cultures were pulsed for the last 16 hours with 1 μCi of [^3^H]TdR and harvested. The suppressive activity of the T_reg _cells can be presented by plotting the percentage of inhibition (100 × (Radioactivity in condition without T_reg _cells – Radioactivity in condition with T_reg _cells)/Radioactivity in condition without T_reg _cells) against the number of T_reg _cells.

### Antibody administration

DBA/1 mice were immunised with CII in CFA; 13 days after immunisation, the mice were treated every second day with 0.25 mg of anti-CD25 (PC61) or control IgG antibodies, for 4 weeks (injected intraperitoneally).

### Histological examination

Forelimbs and hindlimbs were fixed in 10% formalin and decalcified with formic acid (31.5% (v/v) formic acid and 13% (w/v) sodium citrate). The paraffin sections were stained with haematoxylin and eosin.

### Measurement of serum anti-CII antibodies

Blood samples were taken from the orbital sinus and were allowed to clot at room temperature for about 1 hour, and at 4°C overnight. Individual sera were tested by ELISA for antibodies directed against chicken CII. In brief, ELISA plates (Maxisorb; Nunc, Wiesbaden, Germany) were coated overnight at 4°C with native CII (1 μg/ml; 100 μl per well) in coating buffer (50 mM Tris-HCl, pH 8.5, 0.154 mM NaCl), followed by incubation for 2 hours with blocking buffer (50 mM Tris-HCl, pH 7.4, 0.154 mM NaCl and 0.1% caseine) to saturate non-specific binding sites. Serial twofold dilutions of the sera in assay buffer (50 mM Tris-HCl, pH 7.4, 154 mM NaCl and 0.05% Tween 20) were added and incubated for 2 hours at room temperature. The plates were then incubated for 2 hours with peroxidase-conjugated goat anti-mouse IgG (Jackson ImmunoResearch Laboratories, West Grove, PA, USA). Finally, the substrate 3,3',5,5'-tetramethylbenzidine (Sigma-Aldrich) in reaction buffer (100 mM sodium acetate/citric acid, pH 4.9) was added for a 10 min incubation and absorbance was determined at 450 nm. Plates were washed five times between each step with PBS containing 0.05% Tween 20. A serial twofold dilution series of a purified standard was included to permit a calculation of the antibody content of each sample. The standard was purified by affinity chromatography from pooled sera obtained from various arthritic wild-type and IFN-γR KO mice.

### Quantitative RT-PCR

Isolated CD4^+^CD25^+ ^and CD4^+^CD25^- ^cells were pelleted and directly used for total RNA isolation, using the Micro-to-Midi Total RNA Purification System (Invitrogen Life Technologies, Carlsbad, CA, USA). Total RNA (1 μg) was used for random primed cDNA synthesis with RAV-2 reverse transcriptase (Amersham, Aylesbury, Bucks., UK). The reaction mixture was incubated for 80 min at 42°C and the reverse transcriptase was inactivated by incubating the cDNA samples for 5 min at 95°C.

The cDNA samples were then subjected to real-time quantitative PCR, performed in the ABI prism 7700 sequence detector (Applied Biosystems, Foster City, CA) as previously described [29]. The sequences of the forward (-FW) and reverse (-RV) primers and probes (-TP) for β-actin and Foxp3 were as follows: β-actin-FW, AGA GGG AAA TCG TGC GTG AC; β-actin-RV, CAA TAG TGA TGA CCT GGC CG T; β-actin-TP, CAC TGC CGC ATC CTC TTC CTC CC; Foxp3-FW, CCC AGG AAA GAC AGC AAC CTT; Foxp3-RV, TTC TCA CAA CCA GGC CAC TTG; Foxp3-TP, ATC CTA CCC ACT GCT GGC AAA TGG AGT C; TGF-β-FW, TGA CGT CAC TGG AGT TGT ACG G; TGF-β-RV, GGT TCA TGT CAT GGA TGG TGC; TGF-β-TP, TTC AGC GCT CAC TGC TCT TGT GAC AG. Probes were dual-labelled with 5'-FAM and 3'-TAMRA.

All primers and probes were designed with the assistance of the computer program Primer Express (AB) and were purchased from Eurogentec (Seraing, Belgium). The 5'-nuclease activity of the *Taq *polymerase was used to cleave a nonextendable dual-labelled fluorogenic probe. Fluorescent emission was measured continuously during the PCR reaction. PCR amplifications were performed in a total volume of 25 μl containing 5 μl of cDNA, 12.5 μl of Universal PCR Master Mix, no AmpErase UNG (AB), each primer at 100 to 300 nM, and the corresponding detection probe at 200 nM. Each PCR amplification was performed in triplicate wells under the following conditions: 94°C for 10 min, followed by 40 or 45 cycles at 94°C for 15 s and 60°C for 1 min. cDNA plasmid standards, consisting of purified plasmid DNA specific for each individual target, were used to quantify the target gene in the unknown samples, as described [29]. All results were normalised to β-actin and/or hypoxanthine–guanine phosphoribosyltransferase (HPRT) to compensate for differences in the amount of cDNA in all samples. Results were similar whether β-actin or HPRT was used as the housekeeping gene.

## Results

### Effect of treatment *in vivo *with depleting anti-CD25 antibodies on the development of CIA in wild-type DBA/1 mice

**Figure 1:**
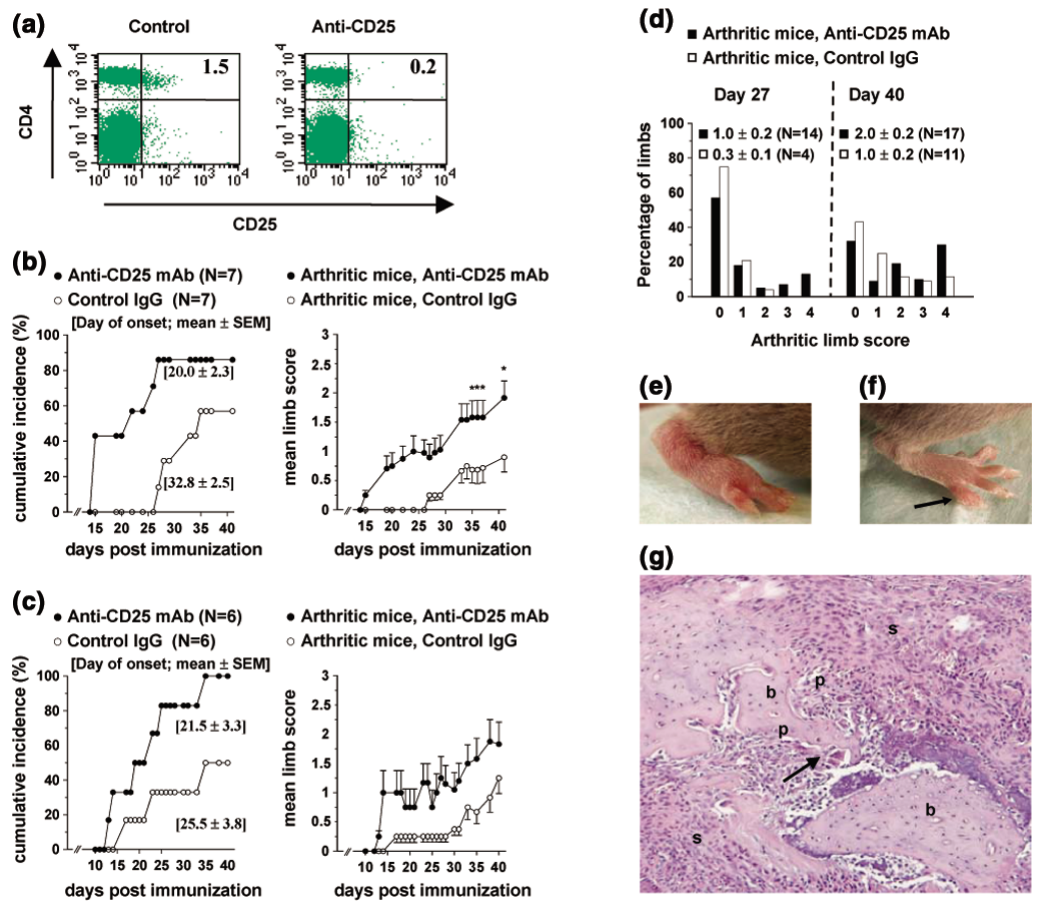
Wild-type mice treated with anti-CD25 antibodies develop a more severe form of arthritis. In three experiments, wild-type DBA/1 mice were immunised on day 0 with collagen type II in complete Freund's adjuvant. From day 11 **(c) **or 13 **(b) **after immunisation onwards, mice were treated every second day with 0.25 mg of depleting anti-CD25 monoclonal antibody (*N *= 7) or with 0.25 mg control rat IgG (*N *= 7). **(a) **Depletion of the CD25^+ ^cell population was checked in the blood twice a week by flow-cytometric analysis with anti-CD4 and anti-CD25 antibodies. A representative staining pattern on day 27 is shown. The percentages of CD4^+^CD25^+ ^cells in control-treated mice (left plot) and anti-CD25-treated mice (right plot) are shown. **(b, c) **Cumulative incidence of arthritis (and mean day of disease onset) and the mean limb score of the arthritic mice in female **(b) **and male **(c) **wild-type mice treated with anti-CD25 or control IgG are shown (the maximum score per limb is 4). Error bars indicate SEM. The data from the female mice are representative of two independent experiments. The data of the three experiments were pooled and the percentage of limbs with each limb score on days 27 and 40 after immunisation is shown in **(d)**. The mean limb score of the arthritic mice in the two groups is also indicated for the two time points and is significantly higher in the treated mice (*P *< 0.05; Mann–Whitney *U*-test) than in those receiving control IgG. **(e, f) **Representative pictures of the most severe case of collagen-induced arthritis on day 25 after immunisation of a mouse treated with anti-CD25 **(e) **and a mouse treated with control IgG **(f)**. **(g) **Haematoxylin-stained paraffin section of the joint of an anti-CD25-treated mouse on day 42 after immunisation. Hyperplasia and infiltration of immunocompetent cells in the synovium (s) and pannus formation (p) that penetrates into the bone (b) can be seen. Note the presence of osteoclast-like multinucleated giant cells (arrow). *P < 0.05 for comparison with control IgG1-treated mice (Mann–Whitney *U*-test).

In a first set of experiments we tested the importance of T_reg _cells in the pathogenesis of CIA by rendering wild-type mice deficient in T_reg _cells by treating the mice with depleting anti-CD25 antibody. Starting from day 11 or 13 after immunisation with CII in CFA, wild-type DBA/1 mice were treated every second day with anti-CD25 antibodies or control IgG. In a first experiment, female mice were chosen because these are only moderately sensitive to CIA [30,31], so that we would be able to detect both increased and decreased disease severity after CD25^+ ^cell depletion. Blood samples were taken at intervals to confirm the depletion of the CD25^+ ^population (Fig. 1a). In control-treated mice, the development of arthritis (day of onset, incidence and mean limb score) was reminiscent of our previously reported findings in which mice received a single immunisation with CII in CFA [20]. In contrast, mice treated with the anti-CD25 antibodies developed a significantly more severe arthritis with a higher incidence and earlier onset than those receiving control IgG1 (Fig. 1b). In fact, the disease course in antibody-treated mice was very similar to that of IFN-γR KO mice [20-22]. The results were confirmed in an additional experiment with female mice. A third experiment was also performed on male animals. The data are plotted in Fig. 1c. Here again, anti-CD25-treated mice developed a higher incidence and a more severe form of arthritis than control-treated mice, whereas the onset of arthritis was not significantly earlier (Fig. 1d). The data from the three experiments were pooled and the percentages of limbs with the different scores from only arthritic mice in the two groups are shown in Fig. 1d. It can be seen that, at an early time point (day 27 after immunisation), the highest scores of arthritis (scores 3 and 4) were already present in anti-CD25-injected mice, but not yet in their control counterparts. On day 40 after immunisation, mice treated with anti-CD25 developed more limbs with a maximum score of 4 than control-treated mice. The mean limb score on the two days for the two groups are indicated and are significantly different (*P *< 0.05, Mann–Whitney *U*-test). The mean number of involved limbs, ± SEM, on day 40 was 2.8 ± 0.2 and 2.2 ± 0.2 for the treated and control mice, respectively (*P *= 0.07; Mann–Whitney *U*-test). Representative pictures of the most severe case of arthritis of anti-CD25-injected and control mice on day 25 after immunisation are shown in Fig. 1e and Fig. 1f, respectively. To ensure that the more severe form of arthritis in the anti-CD25-treated mice was not merely due to oedema, some mice were killed at day 42 for histological evaluation. The presence of hyperplasia and infiltration of immunocompetent cells in the synovium, pannus formation and osteoclast-like multinucleated giant cells confirmed the authenticity of arthritis (Fig. 1g).

On day 35 after immunisation, the titres of collagen-specific antibodies in the sera were determined. No differences in antibody levels in sera of mice treated with anti-CD25 or control IgG could be detected (data not shown).

### Number and phenotype of CD4^+^CD25^+ ^T_reg _cells in IFN-γR KO and wild-type mice

**Figure 2:**
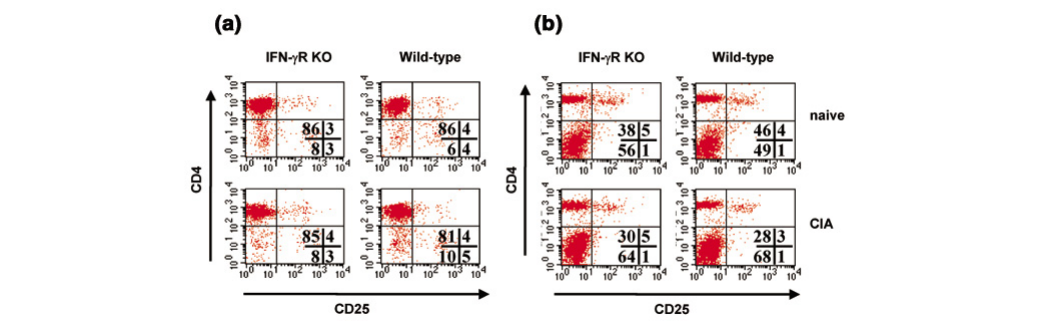
IFN-γ is not required to establish normal numbers of CD4^+^CD25^+ ^T_reg _cells. Thymus cells **(a) **and lymph node cells **(b) **were isolated from IFN-γR KO and wild-type DBA/1 mice, either naive (upper row) or having been immunised 21 days previously with collagen type II in complete Freund's adjuvant (collagen-induced arthritis (CIA), lower row). Cells were stained with anti-CD25-FITC, phycoerythrin-conjugated anti-CD4 and propidium iodide. Dead cells were excluded by gating on propidium iodide-negative cells. The percentages of cells in each quadrant are indicated. Each plot represents a staining pattern of cells from a single female mouse. Identical profiles were observed in male mice. The staining pattern is representative of data obtained in three experiments (Table 1).

To test whether T_reg _cells might be less numerous in IFN-γR KO than in wild-type mice – because this might explain the differences in susceptibility to CIA – we counted CD4^+^CD25^+ ^cells in thymus, lymph nodes and spleen by flow cytometry. IFN-γR KO and wild-type mice were immunised with CII in CFA on day 0. Thymocytes, splenocytes and lymph node cells were obtained on day 21, a time point at which the difference in severity of arthritis between the two groups of mice is most pronounced [20-22]. Groups of naive IFN-γR KO and wild-type mice were also included. A typical CD4/CD25 staining pattern of thymocytes and lymph node cells from IFN-γR KO and wild-type mice is shown in Fig. 2; percentages of CD4^+^CD25^+ ^and CD4^+^CD25^- ^cells are indicated. It can be seen that IFN-γR KO mice did not have smaller proportions of CD4^+^CD25^+ ^cells in the thymus and lymph nodes. Immunised mice, whether wild-type or IFN-γR KO, had rather lower proportions of total CD4^+ ^cells than naive counterparts (for example 31% versus 50% in wild types). However, the real numbers of CD4^+ ^cells per organ were in fact higher after immunisation and did not differ in IFN-γR KO from those in wild-type mice. In fact, the lower percentages of CD4^+ ^cells after immunisation were due to a still larger expansion of the myelopoietic population, a well-recognised phenomenon arising from the use of CFA [22,32].

When over a total of six experiments (Table 1) the numbers of CD4^+^CD25^+ ^cells were expressed as fractions of total CD4^+ ^cell numbers, it appeared that spleens and lymph nodes of IFN-γR KO mice, naive as well as immunised ones, contained slightly higher percentages of CD4^+^CD25^+ ^cells. In spleens and lymph nodes of wild-type mice, 5 to 10% of the CD4^+ ^T cells were CD25^+^, conforming to previously published figures obtained in other mouse strains. Thymuses contained lower percentages of CD4^+^CD25^+ ^cells. A possible explanation might be that thymic CD4^+ ^T cell populations contain not only CD4^+^CD8^- ^but also CD4^+^CD8^+ ^cells, the latter being mostly CD25^-^. In the peripheral lymphoid organs of IFN-γR KO mice, the percentage of CD4^+^CD25^+ ^cells was higher (7 to 14%) than in the wild-type mice (Table 1).

**Figure 3:**
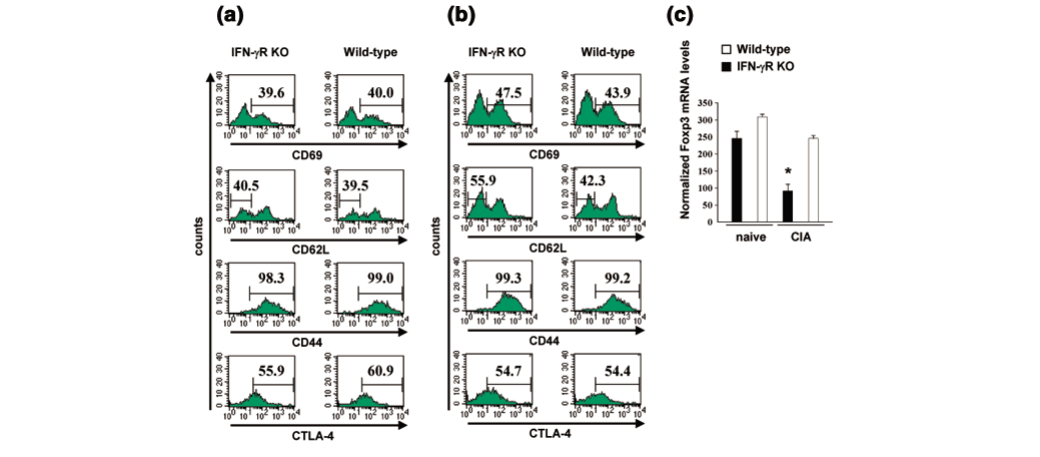
Phenotypic characterisation of CD4^+^CD25^+ ^T cells from immunised IFN-γR KO and wild-type DBA/1 mice. **(a, b) **CD4^+^CD25^+ ^T cells isolated from IFN-γR KO and wild-type mice show a similar expression pattern of activation markers, in naive **(a) **and immunised **(b) **conditions. CD4^+ ^T cells were purified from the lymph node cells of eight IFN-γR KO and wild-type DBA/1 mice, either naive or having been immunised 21 days previously with collagen type II in complete Freund's adjuvant (purity more than 99%). CD4^+ ^T cells were stained for CD25 in combination with CD69, CD62L, CD44 or cytolytic T lymphocyte-associated antigen-4 (CTLA-4). Dead cells were excluded by gating on propidium iodide-negative cells. The numbers represent the percentages of CD4^+^CD25^+ ^cells within the indicated marker. **(c) **Decreased Foxp3 mRNA levels in CD4^+^CD25^+ ^T_reg _cells from immunised mice. Lymph node cells were isolated from eight naive or immunised IFN-γR KO and wild-type DBA/1 mice. Purified CD4^+ ^T cells were stained with anti-CD25-FITC and phycoerythrin-conjugated anti-CD4, and sorted. The purity of the sorted CD4^+^CD25^+ ^population was more than 97%. cDNA samples were prepared from 2 × 10^5 ^cells of each population and were subjected to real-time quantitative PCR analyses. The relative quantity of Foxp3 in each sample was normalised to the quantity of β-actin. Error bars indicate standard error of the means of two (CD4^+^CD24^+ ^cells from naive mice) or three (CD4^+^CD25^+ ^cells from immunised mice) independent experiments. *P < 0.05 for comparison with Foxp3 expression of cells isolated from immunised wild-type mice (Mann–Whitney *U*-test).

Because CD25 is expressed not only by T_reg _cells but also by other recently activated T cells, the slightly higher proportion of CD4^+^CD25^+ ^cells in IFN-γR KO mice is not synonymous with a higher proportion of T_reg _cells. In fact, even a lower proportion of such cells cannot be excluded. We therefore compared the CD4^+^CD25^+ ^T cells from IFN-γR KO and wild-type DBA/1 mice for expression of various other activation markers. Figure 3a,b shows flow-cytometric expression patterns of CD69, CD62L, CD44 and cytolytic T lymphocyte-associated antigen (CTLA-4) in CD4^+^CD25^+ ^T cells from naive and immunised IFN-γR KO and wild-type mice. No major differences in expression levels of these activation markers could be detected between CD4^+^CD25^+ ^T cells from IFN-γR KO mice and those from wild-type mice, whether naive or immunised. Thus, this analysis did not provide evidence for different proportions of any cell type, including T_reg _cells. A specific marker for T_reg _cells is Foxp3. We determined mRNA for this marker by quantitative PCR in CD4^+^CD25^+ ^and CD4^+^CD25^- ^cells, sorted from the lymph node cells of naive or immunised IFN-γR KO and wild-type DBA/1 mice at day 21. In CD4^+^CD25^- ^cells Foxp3 mRNA levels were extremely low (less than 6), and not different between one group of mice and the other. CD4^+^CD25^+ ^cells, in contrast, displayed high expression levels. In cells from naive IFN-γR KO and wild-type mice, levels were comparable. However, CD4^+^CD25^+ ^T cells of immunised IFN-γR KO mice contained levels of Foxp3 that were one-third of those of wild-type mice (Fig. 3c). This lower expression level might be indicative of a smaller proportion of T_reg _cells in the sorted CD4^+^CD25^+ ^cell population or of a lower expression level per cell. To distinguish between these alternatives, a tagging anti-Foxp3 antibody would be needed.

Thus, after immunisation, IFN-γR KO mice possessed a slightly higher percentage of CD4^+^CD25^+ ^cells than wild-type mice. However, the actual T_reg _cells present in this population might be considerably less numerous or might be qualitatively different so as to express less Foxp3.

### Reduced suppressive activity of CD4^+^CD25^+ ^T_reg _cells in arthritic IFN-γR KO mice

**Figure 4:**
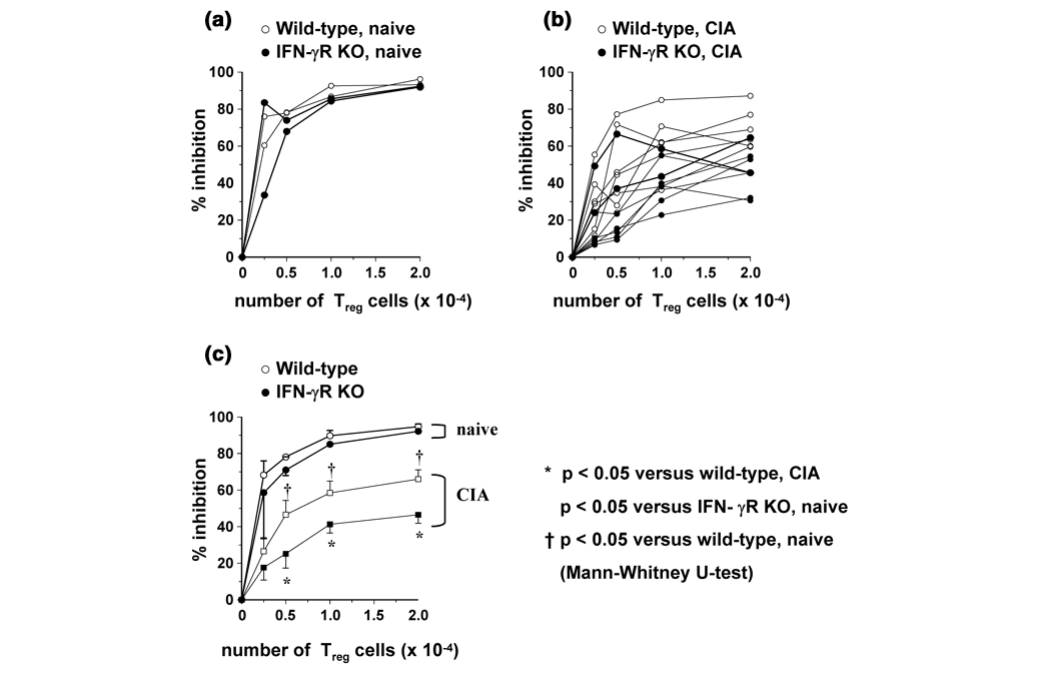
Suppressive capacity of CD4^+^CD25^+ ^cells is decreased more in immunized IFN-γR KO than in wild-type mice. **(a, b) **T_reg _cells, T_eff _cells and accessory cells (ACs) were isolated from lymph nodes and spleen of naive **(a) **IFN-γR KO and wild-type DBA/1 mice or from IFN-γR KO and wild-type DBA/1 mice 21 days after immunisation with collagen type II in complete Freund's adjuvant **(b)**. In each case, a group of seven to nine mice was used. CD4^+^CD25^- ^T_eff _cells (5 × 10^4^) were incubated with anti-CD3 antibody in the presence of ACs and the indicated number of CD4^+^CD25^+ ^T_reg _cells. The percentage inhibition (100 × (Radioactivity in condition without T_reg _cells – Radioactivity in condition with T_reg _cells)/Radioactivity in condition without T_reg _cells) of the proliferation of T_eff _cells (CD4^+^CD25^-^) by increasing numbers of CD4^+^CD25^+ ^T_reg _cells is shown. Two and seven independent experiments are shown in **(a) **and **(b)**, respectively. Each result is the mean of two cups. **(c) **The means of the two (naive mice) or seven (immunised mice) independent experiments shown in **(a) **and **(b)**. Error bars indicate SEM.

To characterise the CD4^+^CD25^+ ^T_reg _cells functionally, we measured their ability to suppress the anti-CD3-induced proliferation of CD4^+^CD25^- ^T_eff _cells *in vitro*. The experiments were performed with CD4^+^CD25^+ ^cells, CD4^+^CD25^- ^cells and ACs. T_reg _suppressive activity was presented by plotting the percentage of inhibition against the number of T_reg _cells. As shown in Fig. 4a,c, the patterns of inhibition in naive IFN-gR KO and wild-type mice were very similar: in both cases 2 × 10^4 ^purified CD4+CD25+ cells were able to inhibit more than 90% of the proliferative response of 5 × 104 T_eff _cells. This result indicates that IFN-γ is not required for T_reg _cells to be able to suppress anti-CD3-induced *in vitro *proliferation.

In a separate set of seven experiments we investigated the suppressive effect of CD4^+^CD25^+ ^cells from mice that had been immunised with CII in CFA. IFN-γR KO and wild-type DBA/1 mice were immunised on day 0, and CD4^+^CD25^+ ^cells, T_eff _cells and ACs were isolated on day 21 after immunisation. The data of the individual experiments are plotted in Fig. 4b and the means of the seven experiments are shown in Fig. 4c. It can be seen that the capacity to suppress TCR-triggered proliferation of T_eff _cells was significantly lower in CD4^+^CD25^+ ^cells isolated from immunised mice than in those of naive animals. Indeed, to obtain 40% inhibition of proliferation, 4.5 × 10^3 ^CD4^+^CD25^+ ^cells from immunised wild-type mice were required, in comparison with only 1.5 × 10^3 ^CD4^+^CD25^+ ^cells from naive wild-type mice. Moreover, CD4^+^CD25^+ ^cells from immunised IFN-γR KO mice were significantly less suppressive than those of immunised wild-type mice: 10^4 ^CD4^+^CD25^+ ^cells were necessary to decrease T_eff _cell proliferation by 40%. In an additional experiment we verified whether the deficit in inhibition by CD4^+^CD25^+ ^cells from immunised IFN-γR KO mice could be corrected by adding excess CD4^+^CD25^+ ^cells. However, with 2 × 10^4 ^and 4 × 10^4 ^CD4^+^CD25^+ ^cells the inhibition on T cell proliferation was 64.6% and 65.8%, respectively, indicating that a plateau level of suppressive activity had been reached.

### Normal levels of TGF-β in IFN-γR KO and wild-type mice

Several studies have shown the critical role of TGF-β in the induction of Foxp3 and the activity of T_reg _cells [10,33,34]. Because IFN-γ and TGF-β act antagonistically with each other (reviewed in [35]), it is possible that TGF-β is upregulated in wild-type mice as a homeostatic response to IFN-γ produced by their activated T cells, and similarly in IFN-γR KO mice the decreased Foxp3 levels and the decreased suppressive activity of T_reg _cells might be due to inadequate amounts of TGF-β produced in the co-cultures or *in vivo *in mice. We therefore analysed the expression of TGF-β by quantitative PCR in T_reg _cells as well as in co-cultures and in spleens of naive and immunised mice. The following results were obtained. First, the levels of TGF-β from the sorted CD4^+^CD25^+ ^cells from immunised IFN-γR KO mice were not different from those of wild-type mice (normalised TGF-β mRNA levels were 179 ± 16 and 193 ± 22, respectively; mean ± SEM for three measurements). Second, because TGF-β might be produced by ACs (or T_eff _cells), quantitative PCR was performed on cells obtained from co-cultures (T_reg _plus T_eff _plus ACs) from immunised IFN-γR KO and wild-type mice. It was found that the levels of TGF-β were even increased in IFN-γR KO cells in comparison with wild-type cells (2,184 versus 1,574, respectively, in the condition of 2 × 10^4 ^T_reg _cells, in a pool of eight mice). Third, the TGF-β levels were also analysed *ex vivo*; that is, in spleen tissue from IFN-γR KO and wild-type mice at day 21 after immunisation (thus at a time point at which T_reg_, T_eff _and ACs were isolated). Here again, the TGF-β levels were found to be slightly increased in spleens from IFN-γR KO mice (816 ± 129 and 633 ± 40 for IFN-γR KO and wild-type mice, respectively). If these results are taken together, the defective activity of T_reg _cells from arthritic IFN-γR KO mice (in comparison with those from wild-type animals) seems not to be associated with a defective TGF-β production.

It was notable that the TGF-β levels were higher in immunised mice than in their naive counterparts (for example, 633 ± 40 and 205 ± 19 for immunised and naive wild-type mice, respectively). These data suggest that the differences in suppressive activity of T_reg _cells from immunised versus naive mice cannot be explained by differences in the TGF-β production.

### T_reg _cells from immunised IFN-γR KO mice have the capacity to inhibit proliferation responses

**Figure 5:**
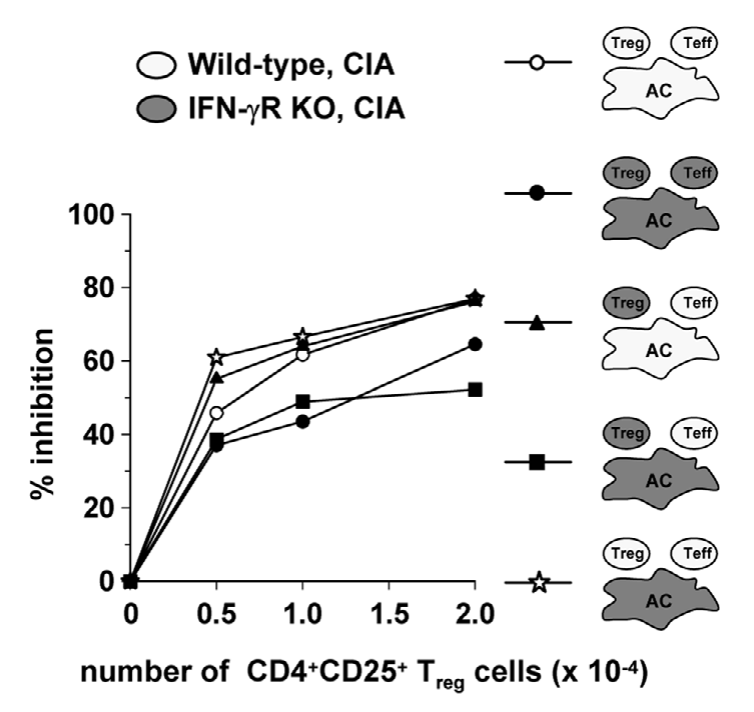
Accessory cells (ACs) of immunised IFN-γR KO mice are required for their defective T_reg _activity. T_reg _cells, T_eff _cells and ACs were isolated from lymph nodes and spleen of IFN-γR KO and wild-type DBA/1 mice 21 days after immunisation with collagen type II in complete Freund's adjuvant. Mixing experiments were performed as indicated. In each set, 5 × 10^4 ^CD4^+^CD25^- ^T_eff _cells were incubated with anti-CD3 antibody in the presence of ACs and the indicated number of CD4^+^CD25^+ ^T_reg _cells. The percentage inhibition of the proliferation of T_eff _cells (CD4^+^CD25^-^) by increasing numbers of CD4^+^CD25^+ ^T_reg _cells is shown. The results are representative of two independent experiments.

We next investigated whether the lower capacity of CD4^+^CD25^+ ^cells from IFN-γR KO mice to downregulate proliferation responses is due to an intrinsic defect or to an altered activity of surrounding ACs and T_eff _cells. We measured the inhibition of anti-CD3-induced proliferation in co-cultures differently reconstituted of CD4^+^CD25^+^, CD4^+^CD25^- ^and ACs, derived either from the same or from different immunised wild-type or immunised IFN-γR KO mice. The combinations tested are indicated in Fig. 5.

As expected, when all cells in the reconstituted co-cultures were of IFN-γR KO mouse origin, suppressive activity was less than when all cells were of wild-type origin. In co-cultures of mixed composition, suppressive activity of IFN-γR KO-derived CD4^+^CD25^+ ^cells was less than that of the wild type only when ACs were from IFN-γR KO origin, but not when they were of wild-type origin. However, such ACs of IFN-γR KO mice were unable to reduce the suppressive effect of wild-type T_reg _cells against wild-type or IFN-γR KO (data not shown) T_eff _cells. These data demonstrate that the defect in inhibiting CD4^+^CD25^- ^T_eff _cells acquired the presence of T_reg _cells from immunised IFN-γR KO mice in combination with their autologous ACs.

## Discussion

We and others have previously demonstrated that IFN-γ(R) KO mice show an accelerated and more severe from of arthritis than their wild-type counterparts, indicating that endogenous IFN-γ acts as a protective factor in CIA [20,21,24,25]. Because CIA has been defined as a Th1-driven disease (reviewed in [17]), the protective effect of IFN-γ in CIA constitutes an enigma that compromises the Th1/Th2 paradigm as a basis for explaining the regulation of autoimmune diseases. A clue to the enigma seemed to be the use of CFA in the induction procedure of CIA. In the absence of IFN-γ, CFA induces an extensive extramedullary myelopoiesis that goes together with an even more pronounced Th1 cytokine profile than in wild-type counterparts [22,36]. The data suggest that IFN-γ can, under certain circumstances, be a strong Th2 inducer, a finding that has recently been confirmed by others [37]. Here, we tested the hypothesis that this protective action of IFN-γ is due to a stimulatory effect on T_reg _cells. Specifically, we addressed the following two questions. Are T_reg _cells important in modulating CIA? And, because we found that depletion of T_reg _cells in wild-type mice increased the severity of CIA, can the higher susceptibility of IFN-γR KO mice to CIA be explained by defects in the number or function of their T_reg _cells?

As to the first question, we found that administration of a T_reg _cell-depleting anti-CD25 antibody to wild-type DBA/1 mice after CFA-assisted immunisation with CII resulted in accelerated and more severe arthritis. In fact, the disease course in these mice was comparable to that in IFN-γR KO mice [20-23]. The actual depletion of T_reg _cells was monitored by flow cytometry, and the authenticity of arthritis was verified histopathologically. These results are in line with those of Morgan and colleagues [26], who showed that the administration of depleting anti-CD25 antibody before immunisation (days –28, –24, –21 and –14) hastened the onset of severe CIA. Because in our experiments antibodies were administered starting from day 11 or day 13 after immunisation, we can conclude that T_reg _cells are important in the pathogenesis of CIA, not only in the immunisation phase but also in the effector phase. In contrast to the findings of Morgan and colleagues [26], the accelerated and more severe course of arthritis was, in our experiments, not accompanied by a higher concentration of anti-collagen II antibodies, possibly due to the different regimen of anti-CD25 treatment. Indirect evidence for the involvement of T_reg _cells in the pathogenesis of CIA comes from data of Min and colleagues [38]. They found that the immune tolerance induced by oral feeding of CII before induction of CIA was mediated by IL-10-producing CD4^+^CD25^+ ^T cells. Notably, in proteoglycan-induced arthritis, another model of autoimmune arthritis, it has been shown that CD4^+^CD25^+ ^T_reg _cells might not have a critical role [39]. This might result from the use of a different auto-antigen.

To address the second question, we compared CD4^+^CD25^+ ^cell numbers and T_reg _cell function in IFN-γR KO DBA/1 mice with those in wild-type mice. According to our hypothesis we expected numbers of T_reg _cells in IFN-γR KO mice to be lower. Counter to this expectation, in each of the six experiments done, we found a trend for a higher proportion of CD4^+^CD25^+ ^T cells in the total CD4^+ ^cell population. This was true for thymic, splenic and lymph node CD4^+ ^cells, in both naive and immunised mice. Analysis of all data as one set revealed a significant difference of about 30% and 20% in naive and immunised mice, respectively. CD25 is not an exclusive marker of T_reg _cells: especially in immunised mice, part of the CD4^+^CD25^+ ^population might be effector rather than regulatory T cells [40,41]. Therefore, to exclude the possibility that we were comparing two completely different populations, we performed additional flow-cytometric characterisation studies on pre-sorted CD4^+^CD25^+ ^cells.

Expression of CD44, CD69, CTLA-4 and CD62L in CD4^+^CD25^+ ^cells from IFN-γR KO mice did not differ from expression in cells from corresponding wild-type mice, whether naive or immunised. However, because T_reg _cells display an activated phenotype, activation markers might not be adequate to distinguish T_reg _cells from activated T_eff _cells. According to Fontenot and colleagues [42] a specific marker for T_reg _cells is Foxp3, because it is highly expressed in CD4^+^CD25^+ ^T_reg _cells and is virtually undetectable in both resting and activated T_eff _cells. We examined Foxp3 expression by determining mRNA levels with PCR. After immunisation, CD4^+^CD25^+ ^cells contained lower levels of Foxp3 mRNA than those of their naive counterparts. Moreover, mRNA levels in immunised IFN-γR KO mice were less than one-third of those in their wild-type counterparts, indicating that IFN-γR KO mice have a smaller number of T_reg _cells or that expression of Foxp3 in each T_reg _cell is lower.

Recently, Bruder and colleagues [43] have shown linked expression of neuropilin-1 and Foxp3, thereby identifying neuropilin-1 as a specific surface marker for CD4^+^CD25^+ ^T_reg _cells able to distinguish them from both naive and recently activated CD4^+^CD25^+ ^non-regulatory T cells.

Nishibori and colleagues [44] demonstrated impaired development of T_reg _cells in naive signal transduction and activators of transcription (STAT)-1-deficient mice, associated with an increased susceptibility to autoimmune disease. Because IFN-γ is among the strongest activators of STAT-1, these observations seem to conflict with ours. However, several cytokines, other than IFN-γ, can also activate STAT-1, including IFN-α, IFN-β, IL-6, IL-9, IL-11, oncostatin M, leukaemia inhibitory factor and the chemokines RANTES and macrophage inflammatory protein 1α [45,46].

To determine whether overall T_reg _cell activity would be lower in IFN-γR KO mice, we co-cultured increasing numbers of CD4^+^CD25^+ ^T cells with fixed numbers of CD4^+^CD25^- ^T_eff _cells and ACs in the presence of anti-CD3 antibody. We observed a dose-dependent inhibition of the proliferative responses by CD4^+^CD25^+ ^T_reg _cells. By estimating numbers of CD4^+^CD25^+ ^cells required to attain a selected level of suppression, we could compare suppressive activity in the different groups of mice. In naive mice, the inhibition curves were almost identical, whether the T_reg _cells were derived from wild-type or IFN-γR KO mice, indicating that endogenous IFN-γ is not an important regulator of the function of constitutive CD4^+^CD25^+ ^T_reg _cells. In co-cultures of cells from immunised wild-type mice, the T_reg _suppressive capacity was about one-third of that in those from corresponding naive mice, and a further halving was noted in co-cultures of cells from immunised IFN-γR KO mice.

The observation that immunisation renders T_reg _cells less suppressive is in line with results of Pasare and Medzhitov [47], who found that microbial triggering of the Toll-like receptor (TLR) pathway by lipopolysaccharide or CpG, which are ligands for TLR4 and TLR9, respectively, blocked the suppressive effect of CD4^+^CD25^+ ^T_reg _cells. Because mycobacteria also contain TLR ligands, immunisation with CFA can be expected to affect T_reg _cell activity similarly. The decrease in suppressive activity that takes place after TLR4 or TLR9 triggering was found to be dependent on IL-6 production [47]. It might therefore be of interest to note that in our experiments, IL-6 production was enhanced after exposure to CFA-assisted immunisation, and this effect was even more pronounced in IFN-γR KO mice (P Matthys, unpublished data). This could provide an explanation for the fact that CD25^+ ^T_reg _cells are totally functional before immunisation but lose (part of) their function after immunisation. However, the most important observation is the lower T_reg _suppressive capacity in IFN-γR KO than in wild-type mice after CFA-assisted immunisation, because this supports our hypothesis that the protective effect of endogenous IFN-γ against CIA could be mediated in part by its stimulatory effect on T_reg _cells.

Because the disease is barely detectable in wild-type mice on day 21 after immunisation, we investigated whether the decreased suppressive activity in immunised wild-type mice was further downregulated at a later time point (namely, day 35 after immunisation, when most of the animals show symptoms of arthritis). However, suppressive activity was not further downregulated to the level seen in homogeneous IFN-γR KO co-cultures, but was comparable to that seen in co-cultures from immunised wild-type mice on day 21 after immunisation (maximal inhibition 60%; data not shown). This indicates that the low suppressive activity as evident in immunised IFN-γR KO mice is restricted to conditions under which IFN-γ is abrogated.

The implication is that the CIA immunisation schedule induces a decrease in T_reg _activity and that endogenous IFN-γ largely counteracts this decrease. It therefore becomes important to know by what mechanism, direct or indirect, IFN-γ influences T_reg _cell function. Addition of anti-IFN-γ antibody to the co-cultures failed to affect suppressive activity (data not shown), indicating that the relevant IFN-γ effect takes place *in vivo *before sampling of the T cells. To examine the role of the different cell components, we tested suppressive activity in mixed co-cultures. CD4^+^CD25^+ ^cells from immunised IFN-γR KO mice, confronted with T_eff _cells and ACs from immunised wild-type mice, were not less suppressive than wild-type CD4^+^CD25^+ ^confronted with wild-type or IFN-γR KO T_eff _cells and ACs. This suggests that lower levels of suppression in homogeneous IFN-γR KO cultures result in part from the presence of IFN-γR KO-derived ACs. And, indeed, when CD4^+^CD25^+ ^and T_eff _cells from immunised IFN-γR KO mice were co-cultured with ACs from immunised wild-type mice, suppressive activity was not inhibited. Finally, ACs from IFN-γR KO mice by themselves were unable to downregulate the activity of wild-type T_reg _cells acting on wild-type T_eff _cells. We therefore conclude that the *in vivo *effect of endogenous IFN-γ that accounts for the greater suppressive activity in wild-type mice than in IFN-γR KO mice concerns reprogramming of both ACs and T_reg _cells.

Because CD4^+^CD25^+ ^T cells from immunised IFN-γR KO mice were not less suppressive than those of immunised wild-type mice in co-cultures with T_eff _cells and ACs from immunised wild-type mice, we can refute the proposition that the lower expression of Foxp3 in the CD4^+^CD25^+ ^population from immunised IFN-γR KO mice is due to a smaller proportion of T_reg _cells and a larger number of activated T_eff _cells. Indeed, if the CD4^+^CD25^+ ^population from immunised IFN-γR KO mice contained a higher proportion of activated T_eff _cells, suppression by these CD4^+^CD25^+ ^cells should be lower, irrespective of the origin of the T_eff _cells and ACs. Another argument is that the addition of more CD4^+^CD25^+ ^cells failed to improve suppression in co-cultures of cells from immunised IFN-γR KO mice. Our data are therefore more in line with the proposition of a lower Foxp3 expression level per cell.

Expression of Foxp3 could be downregulated by the interaction of T_reg _cells with ACs. ACs might be source of TGF-β, which has been described to convert naive T cells into CD25^+ ^suppressor cells by inducing Foxp3 expression [48]. Because IFN-γ and TGF-β act antagonistically with each other, the low levels of Foxp3 in arthritic IFN-γR KO mice might be due to inadequate amounts of TGF-β produced by ACs or other cells. However, quantitative PCR performed on isolated T_reg _cells, on cells obtained from co-cultures (T_reg _plus T_eff _plus ACs) and on splenocytes from immunised IFN-γR KO and wild-type mice does not support the concept that the defective activity of T_reg _cells *in vitro *or *in vivo *is due to defects in the production of TGF-β. ACs have also been shown to be able to reverse suppression by CD4^+^CD25^+ ^cells through the GITR/GITR-ligand system [49]. GITR (glucocorticoid-induced tumour necrosis factor receptor) is expressed on CD4^+^CD25^+ ^T cells; GITR-ligand is initially upregulated on activated APCs. It remains to be determined whether this process involves a downregulation of Foxp3 expression. This or a similar mechanism might take place during the interaction of T_reg _cells and ACs from immunised IFN-γR KO mice. Co-cultures with ACs of immunised wild-type mice might possibly normalise Foxp3 expression in the T_reg _cells of immunised IFN-γR KO mice, together with their T_reg _suppressive activity.

## Conclusions

In conclusion, our experiments support a pathogenesis model that ascribes an important role to T_reg _cells as moderators of the disease course in CIA. In particular we show that T_reg _cells fulfil this role not only during the induction phase but also during the effector phase of the autoimmune response. Furthermore, we were able to refine the model by showing that, after the immunisation with CII in CFA, T_reg _cells lose part of their suppressive potential. This effect is more pronounced in IFN-γR KO than in wild-type mice, indicating that, in this system, IFN-γ acts as an upregulator of T_reg _activity, which might be part of the explanation for the well-known protective effect of endogenous IFN-γ. Finally, we present evidence that the mechanism underlying the effect of IFN-γ on T_reg _cell activity is exerted in part via ACs.

## Abbreviations

ACs = accessory cells; CFA = complete Freund's adjuvant; CIA = collagen-induced arthritis; CII = collagen type II; CTLA = cytolytic T lymphocyte-associated antigen; ELISA = enzyme-linked immunosorbent assay; fetal calf serum = FCS; FITC = fluorescein isothiocyanate; GITR = glucocorticoid-induced tumour necrosis factor receptor; IFN-γ = interferon-γ; IFN-γR KO = interferon-γ receptor knock-out; IL = interleukin; MACS = magnetic-activated cell sorting; PBS = phosphate-buffered saline; RT-PCR = reverse transcriptase polymerase chain reaction; STAT = signal transduction and activators of transcription; T_eff _= effector T; TGF = transforming growth factor; TLR = Toll-like receptor; T_reg _= regulatory T.

## Competing interests

The author(s) declare that they have no competing interests.

## Authors' contributions

BDK, HK and TM performed the CIA induction and evaluation. HK, MVB and GL performed the cell purification. DB performed the quantitative PCR. TM and HK performed the flow cytometry. BDK and HK did the *in vitro *experiments. HK, GL and PM designed the study. All authors participated in the interpretation of the data. HK, AB, GL and PM prepared the manuscript. All authors read and approved the final manuscript.

## Figures and Tables

**Table 1:** Proportion of regulatory T cells to the total CD4^+ ^T cell population in lymphoid organs of naive and immunised IFN-γ receptor knock-out and wild-type (WT) DBA/1 mice

			100 × CD4^+^CD25^+^/CD4^+ ^(*N*)	
			
Treatment	Expt no.	Organ	IFN-γR KO	WT
Naive	1	Thymus	3.2 ± 0.6 (5)	2.2 ± 0.7 (5)
		Spleen	10.1 ± 0.9 (3) *	7.4 ± 0.2 (3)
		Lymph nodes	6.9 ± 1.1 (5)	5.1 ± 1.1 (5)
	2	Spleen	14.4 (1)	9.9 (1)
		Lymph nodes	9.1 ± 0.9 (4)	6.6 ± 0.7 (4)
	3	Lymph nodes	11.2 (1)	7.0 (1)
CIA	4	Thymus	3.5 ± 0.9 (3)	4.0 ± 1.6 (3)
		Spleen	11.0 ± 1.3 (2)	7.9 ± 0.7 (2)
		Lymph nodes	10.2 ± 0.8 (6)	7.7 ± 0.6 (6)
	5	Spleen	12.1 ± 2.9 (3)	9.2 ± 0.8 (3)
		Lymph nodes	12.9 ± 1.4 (4)	10.3 ± 1.1 (4)
	6	Lymph nodes	13.4 ± 0.4 (4) *	9.2 ± 0.7 (4)

Cells were obtained from thymuses, spleens or lymph nodes of IFN-γ receptor knock-out (IFN-γR KO) and wild-type DBA/1 mice. In experiments 4 to 6, mice were immunised with collagen type II in complete Freund's adjuvant on day 0, and cells were obtained on day 21 (collagen-induced arthritis; CIA). Cells were stained with anti-CD25-FITC and phycoerythrin-conjugated anti-CD4 antibodies. The proportion of CD4^+^CD25^+ ^in the total CD4^+ ^T cell population is shown. In experiments 1, 2, 4 and 5, *N *(number in parentheses) indicates the number of mice in each experiment; in experiments 3 and 6, *N *represents the number of experiments, each consisting of groups of 5 to 10 mice, from which samples were pooled for analysis. *Significant difference between IFN-γR KO and wild-type mice (*P *< 0.05; Mann–Whitney *U*-test).
